# Population mobility and disease progression in people living with HIV: a machine learning analysis of a 10-year dynamic cohort

**DOI:** 10.3389/fpubh.2026.1856172

**Published:** 2026-06-05

**Authors:** Yu Luo, Hong Li, Xiyue Fan, Lu Liu, Xinglan Tan, Wenting Yang

**Affiliations:** Beibei Center for Disease Control and Prevention, Chongqing, China

**Keywords:** disease progression, HIV/AIDS, loss to follow-up, machine learning (XGBoost/random survival forest models), mobile populations

## Abstract

**Background:**

Inter-regional population mobility poses challenges to the residence-based HIV follow-up system. This study aimed to define source heterogeneity among PLHIV (local incident versus incoming migrant) and to evaluate its predictive value on out-migration (spatial mobility) and its association with disease progression using machine learning models.

**Methods:**

A dynamic longitudinal cohort (*N* = 5,213) was constructed from monthly follow-up data spanning 116 months (2016–2025) in Chongqing, China. An XGBoost model was developed to predict out-migration risk during management, with the SHAP framework introduced for feature contribution analysis. A random survival forest (RSF) model was applied to assess the long-term risk of disease progression (to AIDS stage or death), and partial dependence plots (PDP) were used to dissect the nonlinear associations of core predictors.

**Results:**

The cohort comprised 2,820 local incident cases, 1,606 baseline prevalent cases, and 787 incoming migrants. The XGBoost model achieved an area under the receiver operating characteristic curve (AUC) of 0.849 for predicting out-migration risk; SHAP analysis indicated that the incoming migrant attribute and specific transmission routes (such as injection drug use) were the strongest predictors associated with spatial instability. The RSF model yielded a concordance index (C-index) of 0.7575 for long-term progression risk; Kaplan–Meier curves showed that incoming migrants had a significantly worse survival prognosis than the other groups (log-rank *p* < 0.001). PDP revealed a stepwise risk increment in disease progression after 30 and 50 years of age at diagnosis. Occupation-based stratification indicated the highest predicted progression risk among the Unemployed/Homebound (53.3%) and Agricultural/Migrant/Manual (25.5%) groups.

**Conclusion:**

Incoming migrants exhibited markedly elevated spatial instability and clinical vulnerability. The current management system needs to evolve from static territorial approaches toward cross-regional dynamic collaboration, employing information interoperability and precise stratified interventions to close treatment gaps that arise during mobility.

## 1 Introduction

The widespread use of antiretroviral therapy (ART) has significantly reduced the risk of AIDS-related deaths worldwide, improved the survival outcomes of people living with HIV (PLHIV), and transformed AIDS into a manageable chronic infectious disease (1, 2). Loss to follow-up (LTFU) and delayed care remain major factors hindering HIV prevention and control, affecting the long-term prognosis of PLHIV, and potentially leading to a decline in their overall health and quality of life. Interruptions in antiretroviral therapy increase the risk of HIV transmission (3). Loss to follow-up can easily lead to treatment interruption, which in turn can result in viral rebound, the emergence of drug-resistant variants, and accelerated disease progression; in severe cases, this can lead to death (4). It also hinders the achievement of the “95-95-95” targets set by the UNAIDS (5).

With China’s economic and social development and the acceleration of urbanization, people are concentrating in developed regions and cities in search of better jobs, education, healthcare, and living conditions, resulting in increasingly frequent population mobility (6). In China, the follow-up management of people living with HIV (PLHIV) is primarily based on a local jurisdiction model centered on their current place of residence; however, high rates of population mobility have placed significant pressure on the existing follow-up management system (7, 8). In practice, primary healthcare institutions must not only manage newly diagnosed local HIV-positive individuals but also receive a large number of incoming migrants (i.e., previously confirmed patients) resulting from employment, education, or changes in residence (9). These incoming migrants may be at different stages of disease progression, have varying medical histories, and exhibit significant differences in treatment adherence. The receiving healthcare institutions must re-examine past medical records, assess patients, and adjust treatment regimens, which not only increases the daily workload but also introduces certain risks in clinical management (10, 11). They often face issues such as medical record transfer and regional policy differences during inter-regional migration, thereby increasing the risk of treatment interruption and loss of management.

Numerous studies have been conducted to identify the factors contributing to LTFU or disease progression among PLHIV. Findings vary across regions: studies in Spain indicate that women are at higher risk of loss to follow-up (12), while in South Africa, men are at greater risk (13); a Canadian study found that young people aged 18–34 are more likely to discontinue treatment (14). Among socioeconomic factors, low income (15), unemployment (16), and low educational attainment (17) are all significantly associated with LTFU. HIV-1 subtype and body mass index may influence disease progression (18). Predictive studies on LTFU or disease progression among PLHIV typically aim to identify which patients are at the highest risk of dropping out of care so that services can intervene earlier (5, 19). Static models struggle to capture behavioral changes during long-term follow-up and fail to account for the potential impact of factors such as population mobility on disease prognosis. Conventional survival analysis methods face certain methodological limitations when dealing with high-dimensional data containing complex interactions, as they struggle to effectively identify nonlinear relationships among variables.

This study utilized dynamic follow-up panel data spanning 10 years (2016–2025) from Chongqing, China. The patients included in the study were categorized into three groups based on their origin: baseline prevalent patients, local incident cases, and incoming migrants. The study employed a two-stage machine learning framework: first, an XGBoost model was used to construct an out-migration warning system to identify populations with high-mobility characteristics; second, a random survival forest (RSF) model was applied to assess the long-term risk of disease progression (to AIDS or death) across the different source groups, and partial dependence plots (PDP) were used to analyze the nonlinear associations of key predictors. This study aims to systematically elucidate the predictive value of spatial mobility and source heterogeneity on the dual outcomes in PLHIV. Conceptually, our two-stage framework provides new knowledge by bridging the gap between short-term administrative out-migration and long-term clinical prognosis. By explicitly treating mobility as a predictor, this study provides scientific evidence for optimizing cross-regional collaborative management strategies for mobile populations.

## 2 Materials and methods

### 2.1 Study design and data sources

This study was a retrospective longitudinal observational cohort study. Study data were extracted from the Chinese epidemic information system (a centralized, city-wide networked epidemic data platform) according to current residence coding and comprised continuous monthly follow-up records from January 2016 to December 2025 (116 months in total. Due to a system error, 4 months of actual data are missing.). Monthly discrete cross-sectional data were longitudinally matched using patients’ unique identifier (card number) to reconstruct a dynamic tracking cohort. The study protocol was approved by the Ethics Review Committee of the Chongqing Center for Disease Control and Prevention. The study used de-identified routine surveillance secondary data, and informed consent was waived.

### 2.2 Population classification and variable definitions

To quantitatively assess the predictive value of spatial mobility on the current residence-based management system, this study classified patients into three heterogeneous source groups on the basis of system timestamp logic:

Baseline prevalent patients: patients who were already registered and managed in the local follow-up system at the observation start point (January 2016).

Local incident cases: patients newly added during the follow-up period whose interval between first diagnosis and entry into the local system was ≤ 1 month, representing cases screened locally and managed on-site.

Incoming migrants: patients newly added during the follow-up period whose first diagnosis occurred > 1 month before entry into the local system. The 1-month threshold was strategically selected to account for standard administrative protocols, as the mandatory reporting, confirmatory testing, and local file-establishment processes are typically completed within a 30-day window. To rigorously address concerns regarding potential misclassification bias, a sensitivity analysis was conducted by extending this threshold to 3 months. This group represents individuals carrying prior confirmed medical records who newly migrated into the local management system because of inter-regional mobility and change of residence.

Covariates extracted included age at diagnosis, sex, marital status, education level, transmission route, and occupation. On the basis of the actual data distribution and epidemiological homogeneity (nature of work, expected spatial mobility, and socioeconomic support), occupations were standardized and merged into five categories: Unemployed/Homebound, Agricultural/Migrant/Manual, Commercial/Service, Employed/Student, and Others/Unknown. Missing values for the continuous variable (age) were imputed using the overall median, whereas missing values for categorical variables were grouped into a distinct “Unknown” category to preserve missingness information.

### 2.3 Outcome definitions

The study defined two independent follow-up outcomes:

*Outcome 1*: Out-Migration. This is defined as the formal transfer of patient management jurisdiction from the local follow-up system prior to the end of the observation period. In the real-time registry system, patients who have lost to follow-up (LTFU) at the local level remain in the database, and local deaths are explicitly recorded. Therefore, the disappearance of cases from the local dataset strictly reflects a transfer of jurisdiction across regions due to data access restrictions. This outcome was used primarily to identify high-mobility populations and to evaluate cross-regional loss pressure on jurisdictional medical management.

*Outcome 2*: Disease progression. This was defined as progression to the AIDS stage during follow-up (indicated by CD4 + T cell count < 200 cells/μL or occurrence of definitive opportunistic infections) or all-cause death. For patients who experienced out-migration, right censoring was applied in the survival analysis, with survival time truncated at the month of their last record in the local system.

### 2.4 Statistical analysis and machine learning modeling

The study employed a two-stage algorithmic framework for multidimensional prediction and evaluation.

The first stage focused on out-migration warning. The full sample was randomly divided into training and test sets in a 7:3 ratio, and an XGBoost binary classification model was constructed to predict the probability of patient out-migration. To handle class imbalance, the scale_pos_weight parameter was applied based on the ratio of negative to positive cases. Hyperparameters were predefined (e.g., n_estimators = 300, max_depth = 5, learning_rate = 0.05). A 5-fold cross-validation strategy was employed to ensure model robustness. Model discrimination was assessed with the receiver operating characteristic (ROC) curve and its area under the curve (AUC). Furthermore, the Brier score and calibration curves were used to evaluate model calibration and prediction accuracy. The SHAP (SHapley Additive exPlanations) framework was then introduced to globally quantify and visualize the feature contributions and associations of each baseline feature on the out-migration outcome.

The second stage focused on long-term disease progression. After exclusion of early-censored samples with follow-up observation time ≤ 3 months (to differentiate acute initial administrative loss from actual long-term clinical management failure and to prevent distortion of survival metrics), a Random Survival Forest (RSF) model (hyperparameters: e.g., n_estimators = 200, max_depth = 5) was constructed with survival time and disease progression as the composite outcome. Kaplan–Meier curves were plotted for the different source groups, and the log-rank test was used to evaluate inter-group differences in prognosis. Overall predictive performance of the RSF model was validated with the concordance index (C-index) on the test set. Finally, permutation importance was calculated to rank the long-term prognostic value of each variable, and partial dependence plots (PDP) were drawn to analyze the nonlinear associations of continuous and discrete variables on the risk of disease progression.

All data cleaning, statistical tests, and machine learning modeling were performed in the Python 3.10 software environment. Two-sided *p* < 0.05 was considered statistically significant.

## 3 Results

### 3.1 Cohort baseline characteristics and source distribution

The reconstructed dynamic follow-up cohort ultimately included 5,213 PLHIV. The demographic and clinical baseline characteristics of the cohort, stratified by patient source (2,820 local incident cases, 1,606 baseline prevalent cases, and 787 incoming migrants), are summarized in Table 1.

**Table 1:** Baseline demographic and clinical characteristics of the study cohort, stratified by patient source.

Characteristics	Total cohort (*N* = 5,213)	Local incident cases (*N* = 2,820)	Baseline prevalent cases (*N* = 1,606)	Incoming migrants (*N* = 787)
Gender, *n* (%)				
Male	3,831 (73.5%)	2,037 (72.2%)	1,194 (74.3%)	600 (76.2%)
Female	1,382 (26.5%)	783 (27.8%)	412 (25.7%)	187 (23.8%)
Unknown	0 (0.0%)	0 (0.0%)	0 (0.0%)	0 (0.0%)
Age at diagnosis, years, *n* (%)				
<30	565 (10.8%)	281 (10.0%)	172 (10.7%)	112 (14.2%)
30–50	1,956 (37.5%)	695 (24.6%)	770 (47.9%)	491 (62.4%)
>50	2,692 (51.6%)	1,844 (65.4%)	664 (41.3%)	184 (23.4%)
Marital status, *n* (%)				
Unmarried	1,389 (26.6%)	500 (17.7%)	497 (30.9%)	392 (49.8%)
Married	2,484 (47.6%)	1,587 (56.3%)	682 (42.5%)	215 (27.3%)
Divorced/widowed	1,239 (23.8%)	694 (24.6%)	380 (23.7%)	165 (21.0%)
Unknown	101 (1.9%)	39 (1.4%)	47 (2.9%)	15 (1.9%)
Education level, *n* (%)				
Illiterate	215 (4.1%)	145 (5.1%)	53 (3.3%)	17 (2.2%)
Primary school	1,683 (32.3%)	1,091 (38.7%)	410 (25.5%)	182 (23.1%)
Middle school	1,999 (38.3%)	931 (33.0%)	727 (45.3%)	341 (43.3%)
High school	779 (14.9%)	363 (12.9%)	282 (17.6%)	134 (17.0%)
College+	536 (10.3%)	290 (10.3%)	133 (8.3%)	113 (14.4%)
Unknown	1 (0.0%)	0 (0.0%)	1 (0.1%)	0 (0.0%)
Transmission route, *n* (%)				
Heterosexual	3,393 (65.1%)	2,264 (80.3%)	834 (51.9%)	295 (37.5%)
MSM (men who have sex with men)	644 (12.4%)	286 (10.1%)	206 (12.8%)	152 (19.3%)
IDU (injection drug use)	1,065 (20.4%)	219 (7.8%)	527 (32.8%)	319 (40.5%)
Sex + IDU	56 (1.1%)	14 (0.5%)	25 (1.6%)	17 (2.2%)
Unknown	55 (1.1%)	37 (1.3%)	14 (0.9%)	4 (0.5%)
Occupation group, *n* (%)				
Unemployed/homebound	2,776 (53.3%)	1,536 (54.5%)	844 (52.6%)	396 (50.3%)
Agricultural/migrant/manual	1,328 (25.5%)	784 (27.8%)	391 (24.3%)	153 (19.4%)
Others/unknown	683 (13.1%)	304 (10.8%)	244 (15.2%)	135 (17.2%)
Commercial/service	221 (4.2%)	105 (3.7%)	56 (3.5%)	60 (7.6%)
Employed/student	205 (3.9%)	91 (3.2%)	71 (4.4%)	43 (5.5%)

### 3.2 Prediction and feature analysis of the out-migration outcome

**Figure 1:**
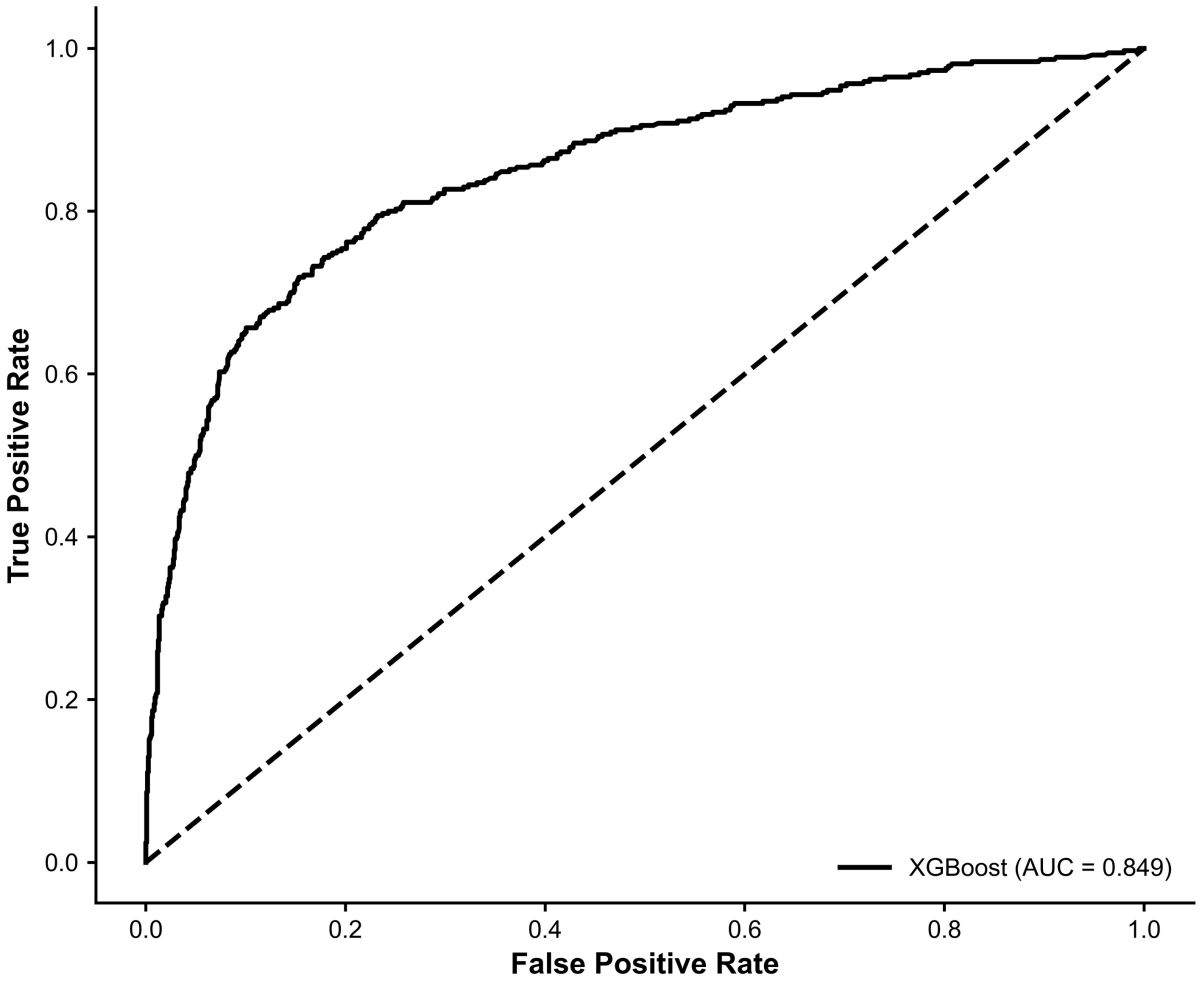
ROC curve for predicting out-migration.

**Figure 2:**
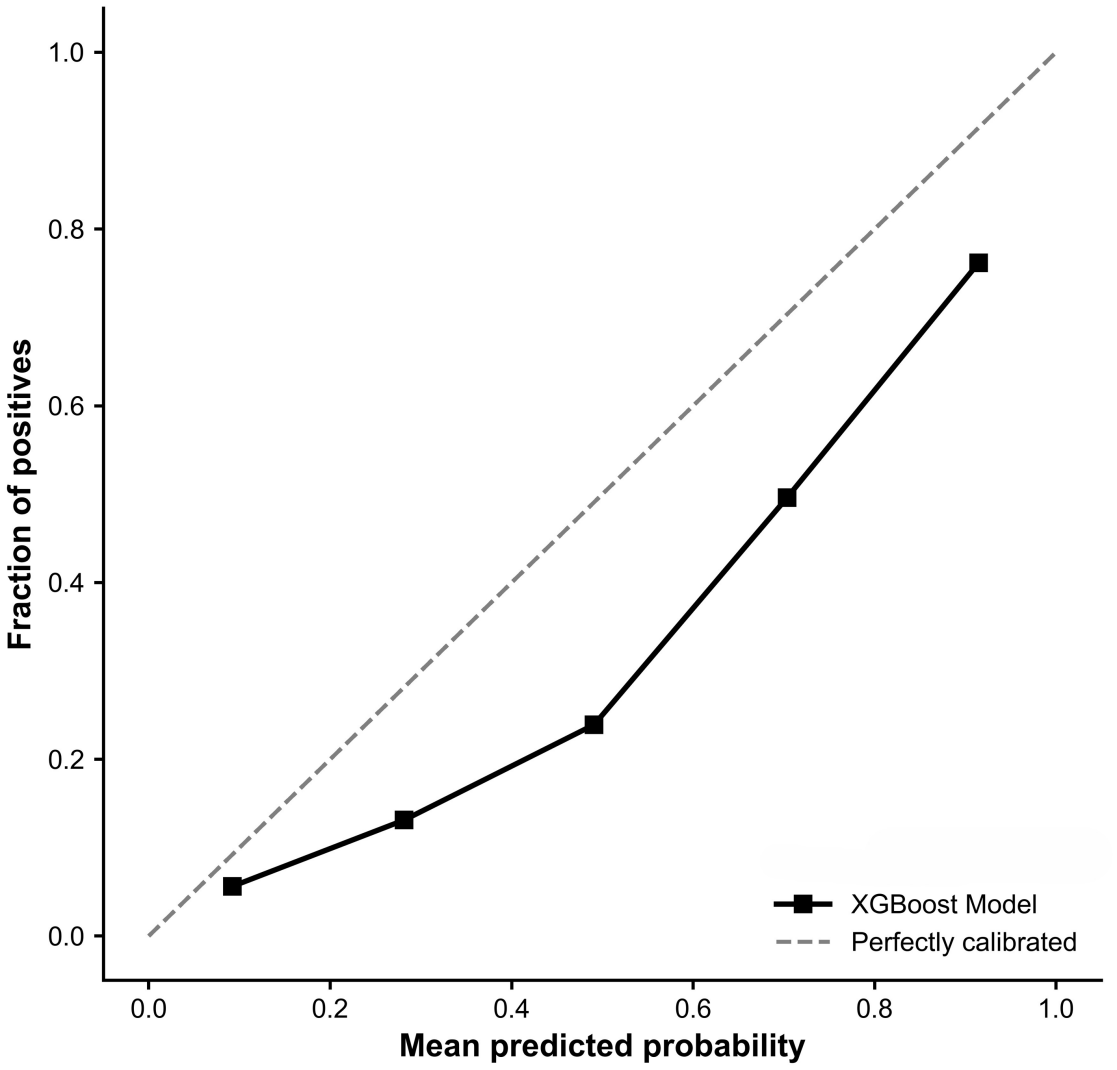
Calibration curve for out-migration model.

For the prediction of out-migration (inter-regional mobility) risk, the constructed XGBoost binary classification model demonstrated good discriminative performance on the test set, with an area under the receiver operating characteristic curve (AUC) of 0.849 (Figure 1). A 5-fold cross-validation confirmed model stability (mean AUC = 0.8104, 95% CI: 0.6711–0.9497). The model also exhibited good calibration accuracy, with a Brier score of 0.1370 (Figure 2).

**Figure 3:**
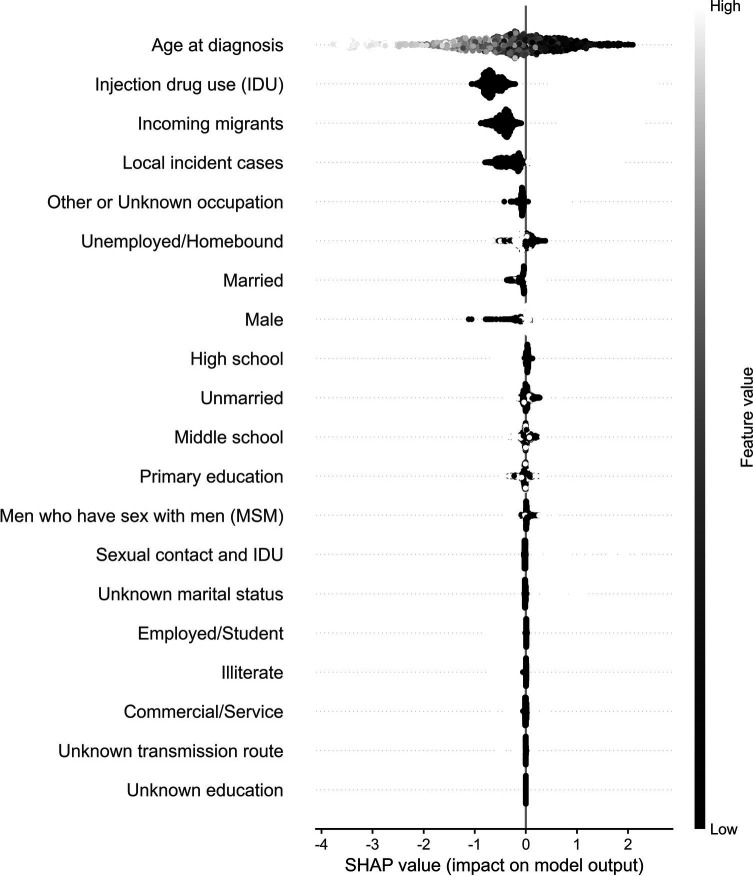
SHAP summary plot for out-migration drivers.

Global interpretation of model features through the SHAP framework showed that the incoming migrant source attribute was the strongest predictor of out-migration (Figure 3). In addition, younger age at diagnosis, injection drug use (IDU) as the transmission route, and Unemployed/Homebound occupational status were also associated with higher out-migration propensity.

### 3.3 Dynamic survival analysis of the disease progression outcome

**Figure 4:**
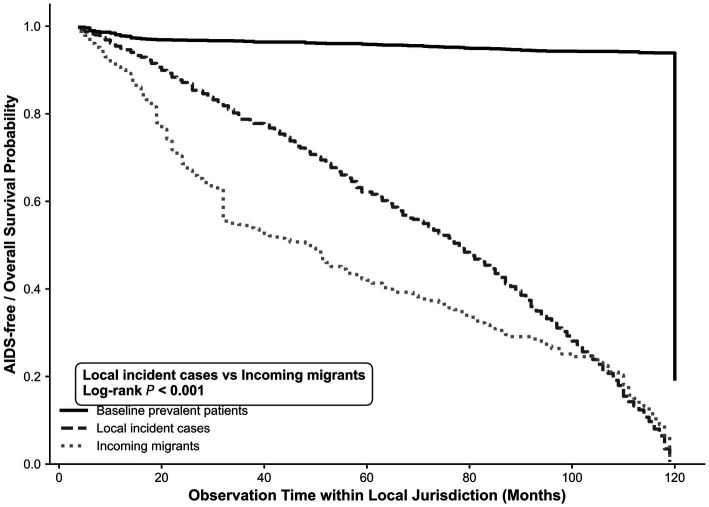
Clinical progression by patient source.

After exclusion of early-censored samples with follow-up time < 3 months, Kaplan–Meier analysis was used to examine differences in the trajectories of disease progression (to AIDS stage or death) across source groups. The survival curves showed that incoming migrants had the steepest decline in event-free survival probability, with significantly worse long-term clinical prognosis than Local incident and baseline prevalent patients (log-rank *p* < 0.001) (Figure 4).

**Figure 5:**
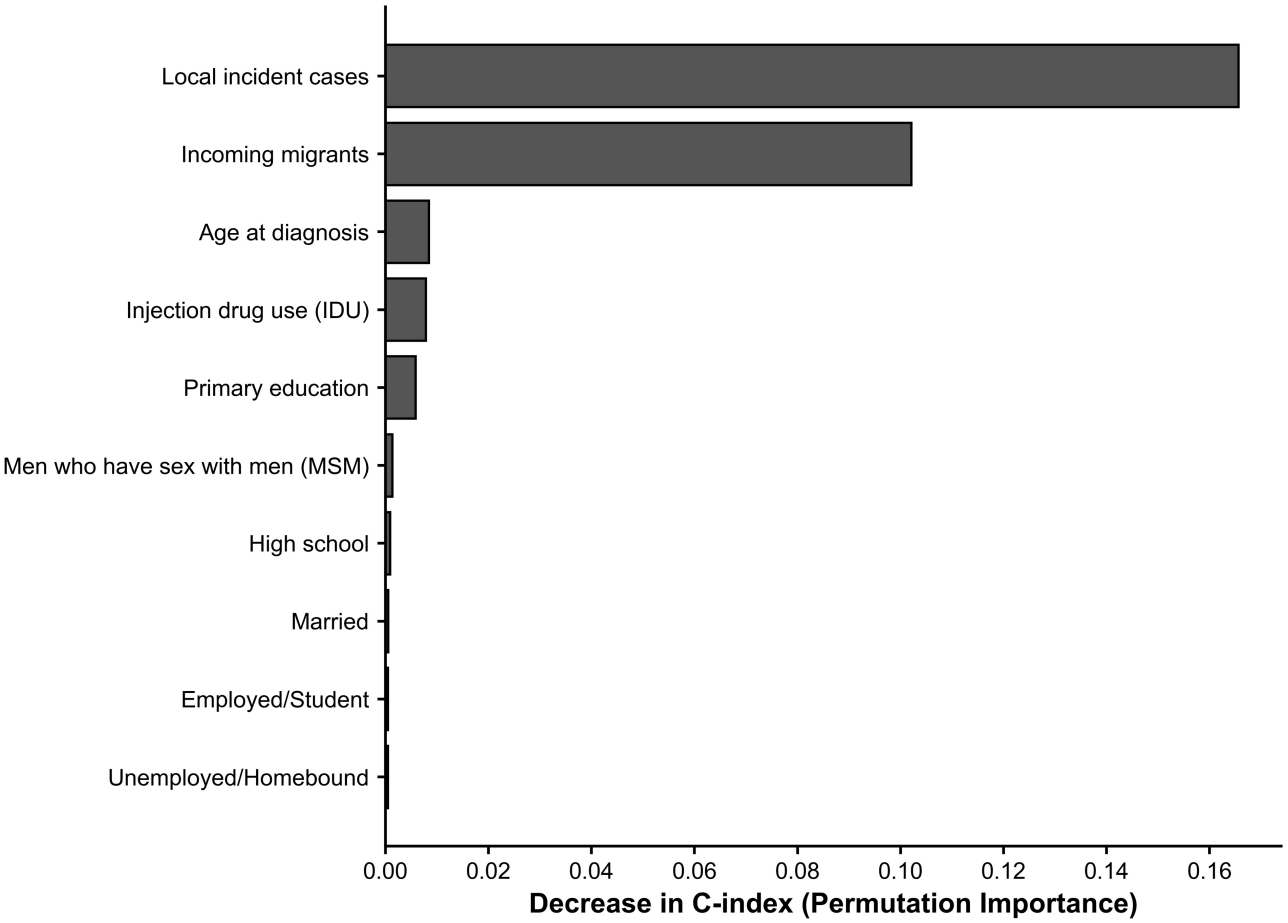
Drivers of clinical progression (RSF).

To comprehensively evaluate the prognostic value of multidimensional features, a random survival forest (RSF) model was constructed; the model achieved a concordance index (C-index) of 0.7575 on the test set, indicating stable predictive performance. Permutation importance analysis confirmed that source attribute (local incident and incoming migrant) and age at diagnosis were the two most important predictors of long-term disease progression (Figure 5).

### 3.4 Nonlinear associations of key predictors and prognostic stratification

**Figure 6:**
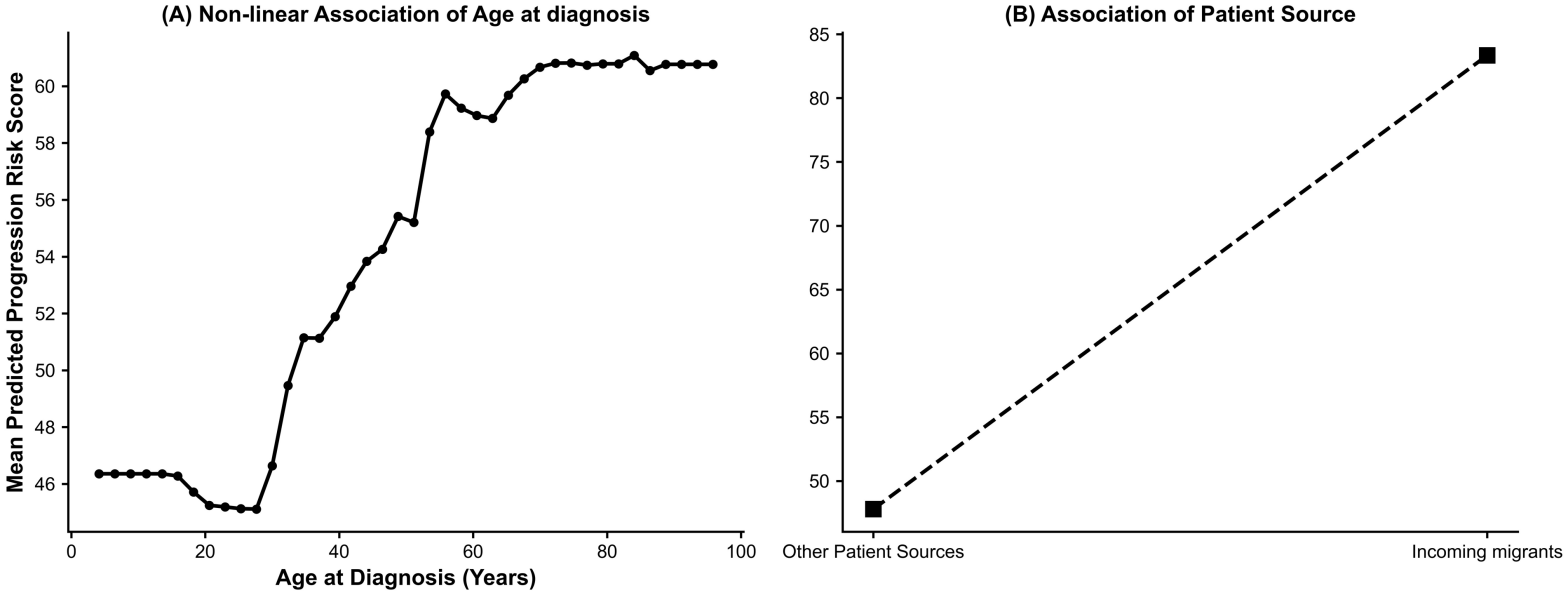
Partial dependence plots for progression risk. **(A)** Non-linear association of mean predicted progression risk score with age at diagnosis, showing a steep increase after thirty years. **(B)** Association of patient source, indicating a higher progression risk score in incoming migrants compared to other patient sources.

Partial dependence plots (PDP) quantified the nonlinear associations of core predictors with the disease progression risk score. Age at diagnosis showed a stepwise increase in associated on disease progression risk, with two clear risk increments after 30 and 50 years of age (Figure 6A). In addition, after adjustment for other variables, the incoming migrant attribute was independently associated with an overall elevation in the prognostic risk score (Figure 6B).

**Figure 7:**
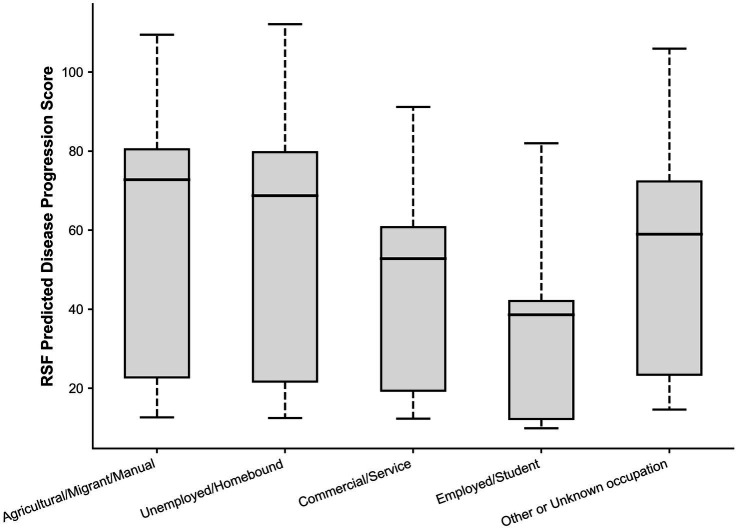
Progression risk stratification by occupation.

Stratification of expected progression risk scores derived from the RSF model revealed significant differences in prognosis across occupational groups (Figure 7). Agricultural/Migrant/Manual and Unemployed/Homebound groups had the highest median expected disease progression risk, whereas the Employed/Student group had the lowest expected risk.

## 4 Discussion

This study used a 10-year real-world dynamic follow-up cohort and a dual-outcome machine learning framework to systematically evaluate the predictive value of source heterogeneity on spatial mobility and its association with long-term prognosis in PLHIV. The results showed that incoming migrants not only faced the highest risk of out-migration but also exhibited significantly faster disease progression during local management compared with local incident cases and baseline prevalent patients. The study found that population mobility has become the norm, yet the current management of people living with HIV remains based on place of residence and relies on a fixed, static management model. This management system may no longer be adequate to meet the prevention and control needs arising from population mobility.

In the prediction of out-migration risk, the XGBoost model and SHAP feature interpretation consistently indicated that a patient’s place of origin is a key factor in determining their likelihood of moving across regions. Most of these individuals have come to the local area for work, education, or relocation, and their overall economic and social foundations are relatively weak (20). Currently, the management of people living with HIV is primarily based on their current place of residence, with static management conducted through household registration and permanent residency status. Due to factors such as varying local policies and standards, as well as inconsistencies in information systems, there remain significant practical obstacles to the transfer of medical records and the continuation of health insurance coverage when people living with HIV move across regions (21). Current management systems are ill-suited to the reality of frequent population mobility; when an infected individual’s medical care arrangements or living conditions change, they are likely to fall out of follow-up care or move across regions again (22). In addition, Unemployed/Homebound occupational status and injection drug use (IDU) as the transmission route also reflect the marginalized position of certain groups within the social support system, further elevating their spatial instability (23).

Survival analysis further highlighted the strong association between this spatial instability into adverse clinical outcomes. Both the RSF prognostic model and Kaplan–Meier trajectories showed that incoming migrant had the highest long-term disease progression risk. Long-term survival of PLHIV depends heavily on the continuity of antiretroviral therapy (ART) (24). Inter-regional mobility is often accompanied by stagnation or delay in care linkages, readily resulting in ART interruption (25). Unlike local incident cases, who can seamlessly enter the local standard treatment network immediately after diagnosis, incoming migrants may have already experienced reduced medication adherence or viral rebound during the migration process before entering local management. This disruption of the cascade of care is likely a key factor associated with the accelerated disease deterioration observed during local follow-up (26).

In addition, the nonlinear risk features extracted by machine learning provide a deeper perspective for refined management. Partial dependence plot (PDP) results showed that the association between age at diagnosis and disease progression risk exhibited a stepwise surge after 50 years of age; this pattern is closely associated with immunosenescence (Immunosenescence) and the accumulation of non-AIDS-defining comorbidities (NADMs) in older PLHIV, underscoring the urgent need for multidisciplinary comprehensive management of aging-infected individuals (27). At the same time, occupation-based risk stratification exposed survival disadvantages among Agricultural/Migrant/Manual and Unemployed/Homebound groups (28, 29). The significant disparities in health outcomes among infected individuals are a classic illustration of how social and health-related factors influence the progression of infectious diseases. This suggests that, in addition to standardized antiviral treatment, the lack of economic and social support resources for infected individuals is also a key factor hindering further improvement in their long-term health.

Based on the findings of the above study, current HIV/AIDS prevention and control efforts need to be adjusted; they can no longer be confined to a static management model based on place of residence, but should instead promote dynamic, cross-regional collaborative management. At the institutional level, efforts should be made to establish regional or national information platforms to facilitate the exchange and sharing of antiretroviral treatment prescription data. Concurrently, procedures related to purchasing medication and medical insurance reimbursement for migrant populations should be streamlined, enabling people living with HIV who are on the move to access treatment and medical insurance services more conveniently, thereby meeting the prevention and control needs arising from the current normalization of population mobility (7). In clinical public health practice, primary follow-up institutions should provide health education as early as possible to newly registered migrants and people living with HIV who are highly mobile, in order to improve their treatment adherence. For individuals who intend to relocate again, primary-level agencies should proactively facilitate cross-agency referrals and ensure seamless coordination of follow-up and treatment services (30, 31). In regions where conditions permit, the application of long-acting injectable antiretrovirals for these populations could be explored to minimize the interference of geographic changes with daily medication (32).

This study has several limitations. Constrained by the variable dimensions available in the routine surveillance database, the models did not incorporate patients’ long-term longitudinal viral load monitoring trajectories or real-time ART adherence records. As a retrospective observational study, unmeasured or uncontrolled confounding factors may exist (for example, patients’ specific income levels or mental health status). although out-migration in our dataset strictly reflects official cross-regional jurisdictional transfer rather than local loss to follow-up, we lose tracking permissions once patients leave the local system. Consequently, we cannot ascertain their subsequent long-term outcomes, such as whether they achieve viral suppression, experience LTFU, or die in their new locations. Patients who migrated out were considered right-censored at the time of their last contact. However, migration is not a random event and is highly correlated with treatment discontinuation and disease progression, suggesting the presence of information-censoring, which may lead to biased survival estimates. Future studies could consider linking multi-source heterogeneous data (such as inter-provincial medical settlement records and pharmacy dispensing data) to enable deeper causal inference.

## 5 Conclusion

This study used a 10-year dynamic follow-up cohort and a dual-outcome machine learning framework to systematically evaluate the independent effects of source heterogeneity on spatial mobility and long-term prognosis in PLHIV. The results showed that incoming migrant not only faced the highest risk of out-migration but also exhibited significantly faster disease progression during local management compared with Local incident and baseline prevalent patients. This finding reveals the structural challenges faced by the current predominantly static current residence-based management system in the context of normalized population mobility.

## Data Availability

The raw data supporting the conclusions of this article will be made available by the authors, without undue reservation.

## References

[1] Oguntibeju OO. Quality of life of people living with HIV and AIDS and antiretroviral therapy. HIV/AIDS. (2012) 4:117–24. doi: 10.2147/HIV.S32321, 22893751 PMC3418767

[2] Cardoso CAA Pinto JA Candiani TMS de CIR Linhares RM Goulart EMA. The impact of highly active antiretroviral therapy on the survival of vertically HIV-infected children and adolescents in Belo Horizonte, Brazil. Mem Inst Oswaldo Cruz. (2012) 107:532–8. doi: 10.1590/S0074-02762012000400014, 22666865

[3] Salimo ZM Avelino-Silva VI da Silva EF Chaves YO Kadri MR d AE Nogueira PA . Factors related to loss to follow-up among people living with HIV: a systematic review. Rev Inst Med Trop Sao Paulo. (2025) 67:e53. doi: 10.1590/S1678-9946202567053, 40834146 PMC12364488

[4] Ergün N Ersan G. Late HIV diagnosis and its impact on immune recovery and clinical outcomes: a retrospective study from Turkey. J Int Assoc Provid AIDS Care. (2025) 24:23259582251404545. doi: 10.1177/23259582251404545, 41342649 PMC12681619

[5] Mushy SE Mtisi E Mboggo E Mkawe S Yahya-Malima KI Ndega J . Predictors of the observed high prevalence of loss to follow-up in ART-experienced adult PLHIV: a retrospective longitudinal cohort study in the Tanga region, Tanzania. BMC Infect Dis. (2023) 23:92. doi: 10.1186/s12879-023-08063-9, 36788523 PMC9926646

[6] Zhang H Chen C Li X. Spatial patterns and determinants of inter-provincial migration across age groups in China. PLoS One. (2025) 20:e0330948. doi: 10.1371/journal.pone.0330948, 40880378 PMC12396665

[7] Taylor BS Garduño LS Reyes EV Valiño R Rojas R Donastorg Y . HIV care for geographically mobile populations. Mt Sinai J Med. (2011) 78:342–51. doi: 10.1002/msj.20255, 21598261 PMC3100665

[8] Camlin CS Charlebois ED. Mobility and its effects on HIV acquisition and treatment engagement: recent theoretical and empirical advances. Curr HIV/AIDS Rep. (2019) 16:314–23. doi: 10.1007/s11904-019-00457-2, 31256348 PMC6663312

[9] Goupil de Bouillé J Pascal C Voyer B Zeggagh J Kherabi Y de Andrade V . How do migrants living with HIV adhere to the HIV care process in high-income countries? A systematic review. BMJ Open. (2025) 15:e093620. doi: 10.1136/bmjopen-2024-093620, 40345690 PMC12067935

[10] Faturiyele I Karletsos D Ntene-Sealiete K Musekiwa A Khabo M Mariti M . Access to HIV care and treatment for migrants between Lesotho and South Africa: a mixed methods study. BMC Public Health. (2018) 18:668. doi: 10.1186/s12889-018-5594-3, 29843667 PMC5975397

[11] Bengtson AM Colvin C Kirwa K Cornell M Lurie MN. Estimating retention in HIV care accounting for clinic transfers using electronic medical records: evidence from a large antiretroviral treatment programme in the western cape, South Africa. Trop Med Int Health. (2020) 25:936–43. doi: 10.1111/tmi.13412, 32406961 PMC8841816

[12] Muga R Egea JM Sanvisens A Arnal J Tural C Tor J . Impact of injecting drug use on the interruption of antiretroviral therapies. J Epidemiol Community Health. (2004) 58:286–7. doi: 10.1136/jech.2003.010066, 15026439 PMC1732746

[13] Kranzer K Lewis JJ Ford N Zeinecker J Orrell C Lawn SD . Treatment interruption in a primary care antiretroviral therapy program in South Africa: cohort analysis of trends and risk factors. JAIDS J Acquired Immune Deficiency Syndromes. (2010) 55:e17. doi: 10.1097/QAI.0b013e3181f275fd, 20827216 PMC3024539

[14] Samji H Taha T Moore D Burchell A Cescon A Cooper C . Predictors of unstructured antiretroviral treatment interruption and resumption among HIV-positive individuals in Canada. HIV Med. (2015) 16:76–87. doi: 10.1111/hiv.12173, 25174373 PMC4300259

[15] Geng EH Bangsberg DR Musinguzi N Emenyonu N Bwana MB Yiannoutsos CT . Understanding reasons for and outcomes of patients lost to follow-up in antiretroviral therapy programs in Africa through a sampling-based approach. JAIDS J Acquired Immune Deficiency Syndromes. (2010) 53:405–11. doi: 10.1097/QAI.0b013e3181b843f0, 19745753 PMC3606953

[16] Ibiloye O Decroo T Eyona N Eze P Agada P. Characteristics and early clinical outcomes of key populations attending comprehensive community-based HIV care: experiences from Nasarawa state, Nigeria. PLoS One. (2018) 13:e0209477. doi: 10.1371/journal.pone.0209477, 30571744 PMC6301656

[17] Agbaji OO Abah IO Falang KD Ebonyi AO Musa J Ugoagwu P . Treatment discontinuation in adult HIV-infected patients on first-line antiretroviral therapy in Nigeria. Curr HIV Res. (2015) 13:184–92. doi: 10.2174/1570162x1303150506181945, 25986369 PMC8538627

[18] Widiyanti M Hadi MI. Viral and host factors are related to the progression of HIV diseases in Mimika, Papua. Open Access Maced J Med Sci. (2019) 7:3429–32. doi: 10.3889/oamjms.2019.437, 32002067 PMC6980796

[19] Ridgway JP Mason JA Friedman EE Oliwa T Flores J Simon J . Comparison of machine learning models to predict loss to follow-up among people with human immunodeficiency virus (HIV). JAMIA open. (2025) 8:ooaf077. doi: 10.1093/jamiaopen/ooaf077, 40709239 PMC12289141

[20] Füller D Vieth SJ Otto B. Migrant and socioeconomic status might intersect in adverse health outcomes. Front Public Health. (2023) 11:1244612. doi: 10.3389/fpubh.2023.1244612, 38094230 PMC10716224

[21] Qin L Chen CP Wang W Chen H. How migrants get integrated in urban China – the impact of health insurance. Soc Sci Med. (2021) 272:113700. doi: 10.1016/j.socscimed.2021.113700, 33497940

[22] Mendelsohn JB Shen Y Xi M Cheng H Bullock S Wu X . Viral suppression by residency status among men living with HIV in the context of the expanded ART policy in Shanghai, China. PLOS Global Public Health. (2026) 6:e0005942. doi: 10.1371/journal.pgph.0005942, 41678551 PMC12900377

[23] Dray-Spira R Persoz A Boufassa F Gueguen A Lert F Allegre T . Employment loss following HIV infection in the era of highly active antiretroviral therapies. Eur J Pub Health. (2006) 16:89–95. doi: 10.1093/eurpub/cki153, 16126745

[24] Ross J Cunningham CO Hanna DB. HIV outcomes among migrants from low- and middle-income countries living in high-income countries: a review of recent evidence. Curr Opin Infect Dis. (2018) 31:25–32. doi: 10.1097/QCO.0000000000000415, 29095720 PMC5750122

[25] Pan Y Horigian VE Saavedra J Alonso E Liao X Botero V . HIV treatment disruption and viral suppression among Venezuelan immigrants living with HIV in Miami: a comparative analysis. AIDS Behav. (2026) 30:368–78. doi: 10.1007/s10461-025-04880-y, 40983786 PMC12929252

[26] Tanser F Bärnighausen T Vandormael A Dobra A. HIV treatment cascade in migrants and mobile populations. Curr Opin HIV AIDS. (2015) 10:430–8. doi: 10.1097/COH.0000000000000192, 26352396

[27] Davis DHJ Smith R Brown A Rice B Yin Z Delpech V. Early diagnosis and treatment of HIV infection: magnitude of benefit on short-term mortality is greatest in older adults. Age Ageing. (2013) 42:520–6. doi: 10.1093/ageing/aft052, 23672932 PMC3684112

[28] Totaro V Patti G Segala FV Laforgia R Raho L Falanga C . HIV-HCV incidence in low-wage agricultural migrant workers living in ghettos in Apulia region, Italy: a multicenter cross sectional study. Viruses. (2023) 15:249. doi: 10.3390/v15010249, 36680288 PMC9861079

[29] Wanni Arachchige Dona S Bohingamu Mudiyanselage S Watts JJ Sweeney R Coghlan B Majmudar I . Added socioeconomic burden of non-communicable disease on HIV/AIDS affected households in the Asia pacific region: a systematic review. Lancet Reg Health. (2021) 9:100111. doi: 10.1016/j.lanwpc.2021.100111, 34327436 PMC8315338

[30] Aldámiz-Echevarria T Fanciulli C Lopez M Perez L Tejerina F Sanchez D . Direct collaboration between hospitals and NGOs, an essential tool to reinforce linkage to care in people living with HIV. Sci Rep. (2025) 15:3583. doi: 10.1038/s41598-025-86540-8, 39875449 PMC11775109

[31] Frederiksen HW Zwisler AD Johnsen SP Öztürk B Lindhardt T Norredam M. Education of migrant and nonmigrant patients is associated with initiation and discontinuation of preventive medications for acute coronary syndrome. J Am Heart Assoc Cardiovasc Cerebrovasc Dis. (2019) 8:e009528. doi: 10.1161/JAHA.118.009528, 31140348 PMC6585379

[32] Brizzi M Pérez SE Michienzi SM Badowski ME. Long-acting injectable antiretroviral therapy: will it change the future of HIV treatment? Ther Adv Infect Dis. (2023) 10:20499361221149773. doi: 10.1177/20499361221149773, 36741193 PMC9893397

